# Long-term probabilistic temperature projections for all locations

**DOI:** 10.1007/s00382-022-06441-8

**Published:** 2022-08-12

**Authors:** Xin Chen, Adrian E. Raftery, David S. Battisti, Peiran R. Liu

**Affiliations:** 1 Department of Statistics, University of Washington, Box 354322, Seattle, WA 98195-4322, USA; 2 Department of Atmospheric Sciences, University of Washington, Box 351640, Seattle, WA 98195-1640, USA; 3 Tower Research Capital, LLC, New York, NY 10013, USA

**Keywords:** Carbon emissions, Coupled model intercomparison project, Intergovernmental panel on climate change, Pattern scaling method, Probabilistic population projections, Natural variability

## Abstract

The climate change projections of the Intergovernmental Panel on Climate Change are based on scenarios for future emissions, but these are not statistically-based and do not have a full probabilistic interpretation. Raftery et al. (Nat Clim Change 7:637–641, 2017) and Liu and Raftery (Commun Earth Environ 2:1–10, 2021) developed probabilistic forecasts for global average temperature change to 2100, but these do not give forecasts for specific parts of the globe. Here we develop a method for probabilistic long-term spatial forecasts of local average annual temperature change, combining the probabilistic global method with a pattern scaling approach. This yields a probability distribution for temperature in any year and any part of the globe in the future. Out-of-sample predictive validation experiments show the method to be well calibrated. Consistent with previous studies, we find that for long-term temperature changes, high latitudes warm more than low latitudes, continents more than oceans, and the Northern Hemisphere more than the Southern Hemisphere, except for the North Atlantic. There is a 5% chance that the temperature change for the Arctic would reach 16 °C. With probability 95%, the temperature of North Africa, West Asia and most of Europe will increase by at least 2 °C. We find that natural variability is a large part of the uncertainty in early years, but this declines so that by 2100 most of the overall uncertainty comes from model uncertainty and uncertainty about future emissions.

## 1 Introduction

The Intergovernmental Panel on Climate Change (IPCC) has issued projections of global temperature change based on four different pathways for emissions and land use up to 2100, each in turn based on a different socioeconomic scenario for the world’s future and designed by a different research group (Van Vuuren et al. 2011; Stocker 2014). However, these projections were not based on a fully statistical approach. Raftery et al. (2017) developed a fully probabilistic approach to forecasting emissions, using a country-specific version of the Kaya identity, and probabilistic forecasts of population, GDP per capita, and carbon intensity (CO_2_ emissions per unit of GDP) for each country. They derived a probabilistic projection of global averaged temperature by convolving the emissions projections with the probabilistic relationship between emissions and average temperature agreed by the IPCC (Pachauri et al. 2014). Liu and Raftery (2021) developed a better method for translating carbon emissions to temperature change based on the Coupled Model Intercomparison Project Phase 5 (CMIP 5) ensemble of climate models (Hurrell et al. 2011), that explicitly accounts for the bias and noise in the models that make up the ensemble.

There has been less work quantifying the probability distribution of local temperature change; see, for example, Schölzel and Hense (2010) for one example for a very limited local area. The IPCC reports contain probability distributions of local climate change to 2100 for each of four prescribed emission scenarios that take into account climate model uncertainty. However, these probability distributions are for 20-year averaged temperature (Houghton et al. 2001; Solomon et al. 2007; Stocker 2014). Since annual averaged temperature is nearly white noise (see below), averaging over a 20-year period reduces the contribution of natural variability by a factor of $\sqrt{20}\sim 4.5$. Similarly, studies have reported on the uncertainty in local climate changes within a single model by performing a large number of simulations and then examining the ensemble average change compared to the spread about the ensemble average, where the former is that model’s forced response and the latter is that model’s representation of natural variability, which often suffers biases compared to the observed natural variability (e.g., Kjellstrom et al. 2011; Hawkins and Sutton 2012; Kay et al. 2015; Hawkins et al. 2016; McKinnon et al. 2017). Also, neither approach takes into account the probability distribution of future emissions. Here, we aim to fill the gap. We develop a fully statistical approach to long-term temperature projections, yielding a probabilistic forecast of local temperature change at any desired location that takes into account uncertainty in population, GDP per capita, energy usage, climate model uncertainty and natural climate variability.

Our method is based on the previous probabilistic forecast of global averaged temperature (Liu and Raftery 2021; Raftery et al. 2017). A typical way to bridge the gap between global temperature and local temperature is to use the pattern scaling method (Santer et al. 1990; Tebaldi and Arblaster 2014). Santer et al. (1990) estimated the spatial features of the externally forced change, standardized by global averaged temperature warming, on the basis of 2xCO_2_ equilibrium simulations using atmospheric models coupled to slab ocean models. Tebaldi and Arblaster (2014) estimated the pattern for global mean temperature change using the simulations from CMIP 5 focusing on multi-model mean patterns, and they verified that the common pattern is independent of the choice of emission scenario. Building on this result, we adopt the estimation method of Tebaldi and Arblaster (2014) based on the CMIP 5 simulations under the RCP 8.5 pathway. We then combine the estimates of patterns for temperature change with the samples of global mean temperature from the Markov chain Monte Carlo (MCMC) estimation of the Bayesian hierarchical model of Liu and Raftery (2021) to obtain the samples of local temperature which approximate its distribution.

In addition to the projections of temperature, it is also important to trace the sources of projection uncertainty. Hawkins and Sutton (2009) decomposed the uncertainty into three sources: internal variation (i.e., unforced temperature variability simulated by the models), climate sensitivity and scenario uncertainty. For the uncertainty scenario, they assumed the three IPCC emission scenarios A1B, A2 and B1 were equally probable. They showed that internal variation was a major contributor to overall prediction uncertainty at first, and that its contribution fell rapidly over time, based on the earlier CMIP 3 ensemble of climate models. However, their method is based on the variance of model predictions rather than the variance of the prediction errors, defined as the differences between predictions and observations, and so ignores the latter part of overall uncertainty. We use the simulations from CMIP 5 and find similar results for most of the local areas, with the prediction error naturally included in our model.

The paper is organized as follows. In Sect. 2, we describe the data we use, and describe the method, giving results of its assessment by out-of-sample predictive validation. In Sect. 3, we show the estimation results of the scaling pattern and natural variability, as well as the projection results of temperature. We conclude in Sect. 4 with a discussion.

## 2 Methods

**Fig. 1:**
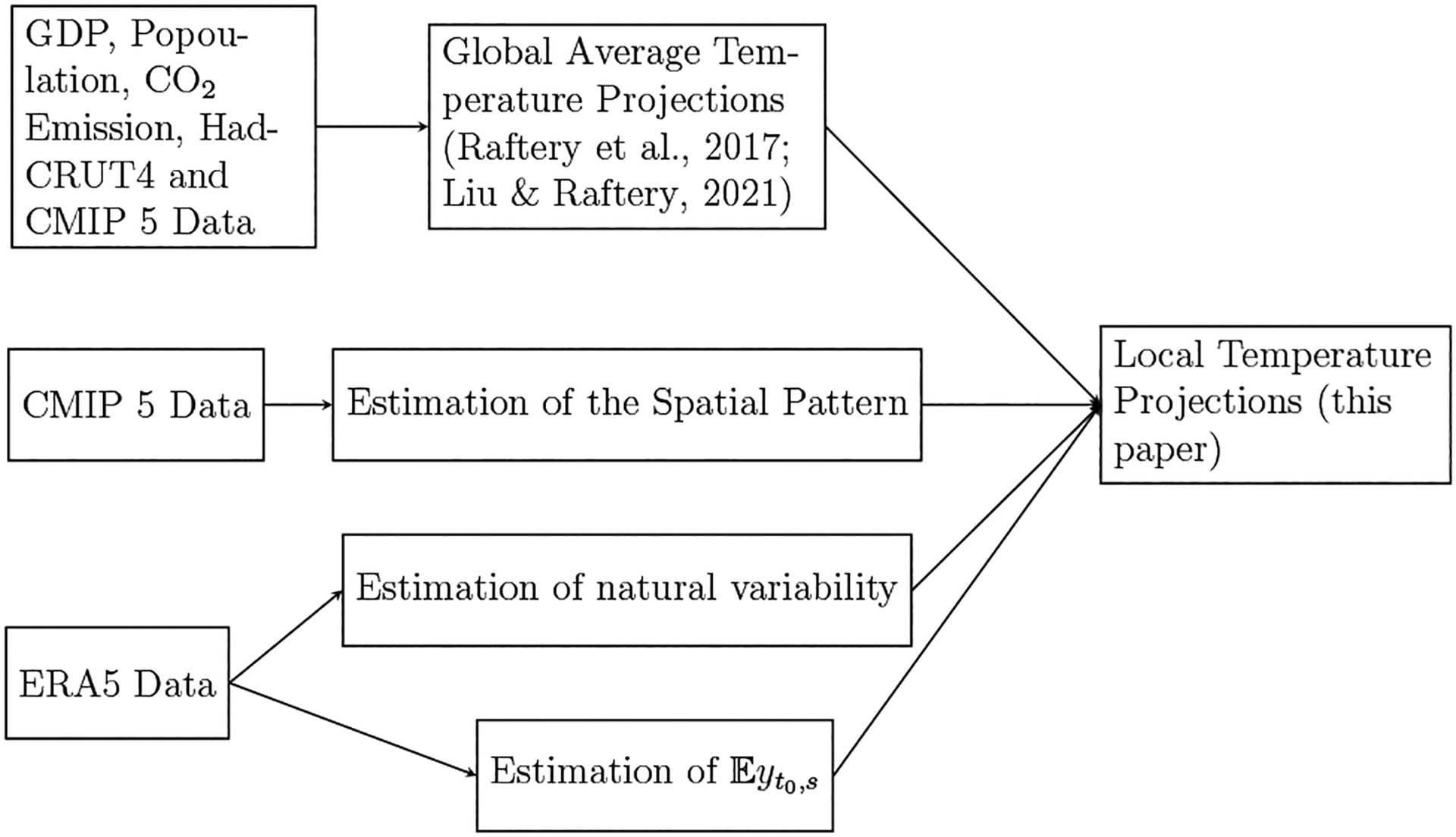
Systems diagram of our method

Our overall method consists of several steps, and these are shown in the systems diagram in Fig. 1. We now describe the different parts of the method.

### 2.1 Data

We produce a probabilistic forecast of CO_2_ emissions using the method of Raftery et al. (2017), which combines probabilistic forecasts of population, GDP per capita, and carbon intensity for most countries. These forecasts are based in turn on past data on these quantities. For population, we use the UN’s 2019 estimates of population for all countries from 1950 to 2015 (United Nations 2019). We include 159 countries with good historic data on all of population, GDP and carbon emissions; these account for 99% of the world’s population. We use probabilistic population projections for each of these countries produced by the model used by the UN (Raftery et al. 2012).

GDP per capita data come from the Maddison Project, 2018 version (Bolt et al. 2018), using data from 1960 to 2015. This uses purchasing power parity (PPP) rather than market exchange rates, and we use real GDP per capita in 2011 US dollars with a 2011 benchmark. Data on CO_2_ emissions come from the Global Carbon Budget (Le Quéré et al. 2018). We use data from 1960 to 2015.

To estimate the common pattern of annual temperature change per °C of global annual-average warming, we use the CMIP 5 model simulations (Taylor et al. 2012). These data include historical simulations back to January 1861, and provide estimates of future temperature up to December 2100 under the RCP 8.5 pathway with the following 35 different CMIP 5 models: ACCESS1-0, ACCESS1-3, bcc-csm1-1, BNU-ESM, CanESM2, CCSM4, CESM1-BGC, CESM1-CAM5, CMCC-CM, CMCC-CMS, CNRM-CM5, CSIRO-Mk3-6-0, EC-EARTH, FGOALS-g2, FIO-ESM, GFDL-CM3, GFDL-ESM2G, GFDL-ESM2M, GISS-E2-H, GISS-E2-R, HadGEM2-AO, HadGEM2-CC, HadGEM2-ES, inmcm4, IPSL-CM5A-LR, IPSL-CM5A-MR, IPSL-CM5B-LR, MIROC5, MIROC-ESM, MIROC-ESM-CHEM, MPI-ESM-LR, MPI-ESM-MR, MRI-CGCM3, NorESM1-M, NorESM1-ME. To forecast the global averaged temperature in each model, we adopt the method of Liu and Raftery (2021).

To estimate the natural variability in local temperature, we use the ERA5 reanalysis dataset (Hersbach et al. 2019), from which we use the air temperature at 2m above the surface of land, sea or inland waters. The data are updated monthly, are available from January 1979 onwards, and are gridded to a regular latitude-longitude grid of 0.25 degrees. We use the annual data from 1979 to 2015, averaging the data to a regular latitude-longitude grid of 2.5 degrees that is commensurate with the climate model output.

### 2.2 Statistical model

#### Model specification

We build the statistical model in three steps. First, we use the statistical model of Raftery et al. (2017) for probabilistic forecasting of CO_2_ emissions. This involves Bayesian hierarchical time series models for fertility and mortality, and hence population, for GDP, and for carbon intensity for each country. Second, we use the model of Liu and Raftery (2021) for global averaged temperature projections. We adopt their Bayesian time series models of the ensemble of CMIP 5 models forecasts, historical global averaged simulations and historical temperature anomalies for estimating the bias and measurement error variance of the CMIP 5 models. We then use the linear relationship between global averaged temperature and cumulative CO_2_ emissions (Stocker 2014; Liu and Raftery 2021), together with the estimated uncertainty and bias of CMIP 5 models to generate global averaged temperature projections. Finally, we take the probabilistic forecast of global averaged temperature as an input and use the pattern scaling method to generate the predictive distribution of future local, annual-averaged temperatures.

#### Pattern scaling models

We use s as the index for grid box and t for year. Altogether we have 72 × 144 = 10,368 grid boxes, so that s∈{1,2,…,10,368}. We denote the local annual-averaged temperature in year t and grid box s by $y_{t,s}$ and the global, annual-averaged temperature in year t by $a_{t}$. We denote by $y_{t,s,i}$ and $a_{t,i}$ the corresponding local temperature and global averaged temperature with respect to a specific CMIP 5 model i. We need this notation because the global averaged temperature projections in Liu and Raftery (2021) are model-specific.

We define two different kinds of patterns for temperature change. The first one is the model-specific pattern, denoted by $b_{s,i}$ which is time-invariant focusing on a specific CMIP 5 model i at grid box s. We call this the *multi-pattern approach*, because it estimates a different spatial pattern of temperature change for each climate model.

The second pattern is denoted by $b_{s}$, focused on the multi-model average at grid box s. We call this the *single-pattern approach* because it uses the same spatial pattern of temperature change for all climate models. The single-pattern approach is often called “pattern scaling” (Tebaldi and Arblaster 2014), while the multi-pattern approach incorporates differences in the pattern of the forced response across CMIP 5 models.

Based on the pattern scaling method and the two approaches, we will use two models, namely:

(1) $$y_{t,s,i}=Ey_{t_{0},s}+b_{s,i}\left(a_{t,i}-a_{t_{0},i}\right)+\varepsilon_{t,s},$$

and

(2) $$y_{t,s}=Ey_{t_{0},s}+b_{s}\left(a_{t}-a_{t_{0}}\right)+\varepsilon_{t,s}.$$

Here $\varepsilon_{t,s}$ is a zero-mean error term that represents the natural variability related to both time and space; it includes the prediction error in a natural way. The symbol E denotes mathematical expectation. We take $t_{0}=2015$ which means we start our projection from 2016.

Note that the models (1) and (2) are both marginal models that aim to produce proabilistic projections of local temperature in individual grid boxes separately, but do not aim to estimate a joint distribution of temperature in different grid boxes. Thus the method is appropriate for projecting temperature in one grid box, but not, for example, the average temperature over a set of grid boxes. To do the latter would require estimating the joint projection distribution (including the correlation) of the temperatures in multiple grid boxes. Ignoring the spatial correlation between different grid boxes should not in principle affect the marginal distribution of temperature in any grid box, since the joint predictive distribution is a multivariate normal (MVN) distribution, and in an MVN distribution estimation of the marginal distribution of one variable (here the temperature in one grid box) is unaffected by the correlation structure (Muirhead 2005). We show later that the resulting projections are indeed of good quality.

Also, note that we do not assume that the outputs of these models are i.i.d. Rather, we use the set of ESMs to represent the range of possible climate sensitivities supported in the literature.

The projections of local temperature are based on the following steps.

#### Global averaged temperature projections

First we produce probabilistic forecasts of the CO_2_ emissions for all countries using the method in Raftery et al. (2017). We produce forecasts of population for all countries, and forecasts of GDP per capita and carbon intensity jointly for all the countries. We then draw samples of future population, and sample jointly from the posterior predictive distribution of GDP per capita and carbon intensity for all future years and countries. We then multiply them together to obtain posterior trajectories of CO_2_ emissions for each country based on Kaya’s identity and then sum the country emissions to obtain the time-evolving probability distribution of global emissions.

We then make forecasts of the global averaged temperature anomaly for each CMIP 5 model based on our predicted CO_2_ emissions by using the linear regression model of Liu and Raftery (2021). For each emission trajectory, we calculate the global cumulative emissions from 2015 and multiply them by a coefficient specific to a CMIP 5 model that maps the global averaged temperature to the cumulative CO_2_ emissions (Liu and Raftery 2021), which is estimated by the linear regression model.

Finally, we use the dynamic time series model of Liu and Raftery (2021) to forecast the bias and uncertainty of the historical CMIP 5 estimates, and we use these forecasts to correct the temperature anomaly forecast for each trajectory. All the models are fitted using Markov Chain Monte Carlo (MCMC) sampling, as implemented in the *rjags* package in the R programming language (R Core Team 2021).

#### Estimating the spatial pattern of temperature change

We define two different patterns which are estimated by two different methods, the so-called “multi-pattern” $\left(b_{s,i}\right)$ and “single-pattern” $\left(b_{s}\right)$ methods. Both of the estimation procedures involve only the CMIP 5 local temperature simulations.

#### Multi-pattern method

We denote by ${\tilde{y}}_{t,s,i}$ the CMIP 5 local temperature simulations for year t, grid box s and CMIP 5 model i. This value can be either a historical simulation or a projection of future temperature by CMIP 5. The multi-pattern method is based on Eq. (1). We use a weighted average to estimate the corresponding global averaged temperature for year t, CMIP 5 model i, which is denoted by ${\tilde{a}}_{t,i}$. For ${\tilde{y}}_{t,s,i}$, the weight is defined by the cosine of the latitude of grid box s, that is, $\text{cos}\left(lat_{s}\right)$. Specifically,

(3) $${\tilde{a}}_{t,i}≔\frac{\sum_{s=1}^{72\text{*}144}\text{cos}\left({\text{lat}}_{s}\right){\tilde{y}}_{t,s,i}}{\sum_{s=1}^{72\text{*}144}\text{cos}\left(lat_{s}\right)}.$$

Define

(4) $${\tilde{y}}_{2080:2099,s,i}≔\frac{{\tilde{y}}_{2080,s,i}+\cdots +{\tilde{y}}_{2099,s,i}}{20},\;{\tilde{y}}_{1980:1999,s,i}≔\frac{{\tilde{y}}_{1980,s,i}+\cdots +{\tilde{y}}_{1999,s,i}}{20}$$

(5) $${\tilde{a}}_{2080:2099,i}≔\frac{{\tilde{a}}_{2080,i}+\cdots +{\tilde{a}}_{2099,i}}{20},\;{\tilde{a}}_{1980:1999,i}≔\frac{{\tilde{a}}_{1980,i}+\cdots +{\tilde{a}}_{1999,i}}{20}$$

where ${\tilde{y}}_{2080:2099,s,i}$ and ${\tilde{y}}_{1980:1999,s,i}$ denote the averages of local annual temperature at grid box s, model i for 2080–2099 and 1980–1999 from CMIP 5 simulations. Similarly, ${\tilde{a}}_{2080:2099,i}$ and ${\tilde{a}}_{1980:1999,i}$ denote the corresponding global, annual-averaged temperatures over the respective 20 year periods. The multi-pattern estimate is then

(6) $${\hat{b}}_{s,i}=\frac{{\tilde{y}}_{2080:2099,s,i}-{\tilde{y}}_{1980:1999,s,i}}{{\tilde{a}}_{2080:2099,i}-{\tilde{a}}_{1980:1999,i}},$$

which is the difference between the local average temperature of the late 21st century and the late 20th century standardized by the corresponding difference of the global average temperature.

#### Single-pattern method (pattern scaling)

The single pattern is estimated as a multi-model average based on Eq. (2). Instead of using a specific model as in the multi-pattern method, we estimate the local average temperature, global average temperature and patterns by averaging over all the 35 CMIP 5 models. Our estimate is as follows:

(7) $${\tilde{b}}_{s}=\frac{{\tilde{y}}_{2080:2099,s}-{\tilde{y}}_{1980:1999,s}}{{\tilde{a}}_{2080:2099}-{\tilde{a}}_{1980:1999}},$$

Where

(8) $${\tilde{y}}_{2080:2099,s}≔\frac{\sum_{i=1}^{35}\sum_{t=2080}^{2099}{\tilde{y}}_{t,s,i}}{35\;\text{*}\;20},\;{\tilde{y}}_{1980:1999,s}≔\frac{\sum_{i=1}^{35}\sum_{t=1980}^{1999}{\tilde{y}}_{t,s,i}}{35\;\text{*}\;20},$$

(9) $${\tilde{a}}_{2080:2099}≔\frac{{\tilde{a}}_{2080}+\cdots +{\tilde{a}}_{2099}}{20},\;{\tilde{a}}_{1980:1999}≔\frac{{\tilde{a}}_{1980}+\cdots +{\tilde{a}}_{1999}}{20},$$

and

(10) $${\tilde{a}}_{t}≔\frac{\sum_{i=1}^{35}\sum_{s=1}^{72\text{*}144}\text{cos}\left({\text{lat}}_{s}\right){\tilde{y}}_{t,s,i}}{35\sum_{s=1}^{72\text{*}144}\text{cos}\left(lat_{s}\right)}.$$

#### Estimation of natural variability

**Fig. 2:**
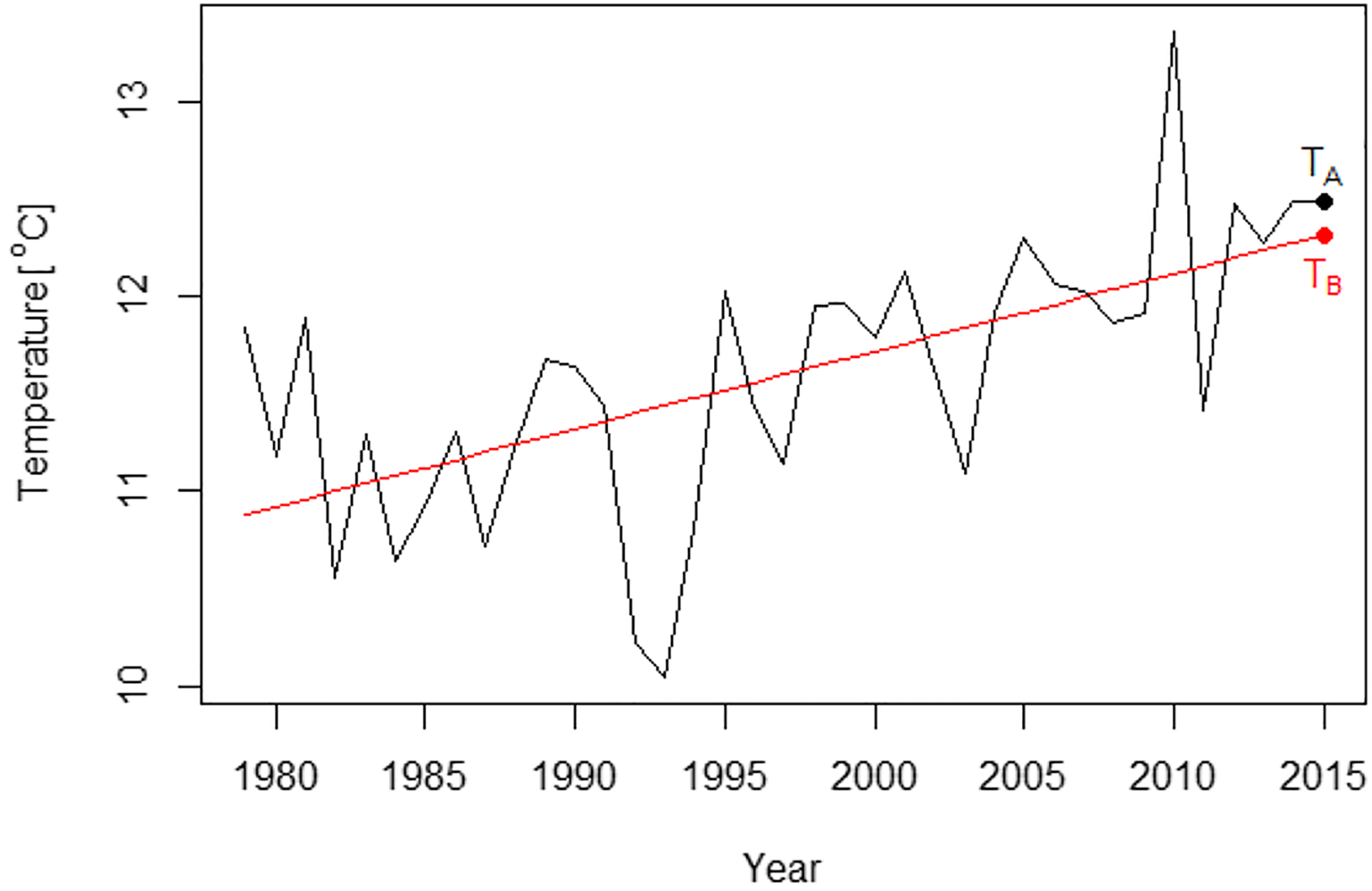
Historical temperature values and linear trend for one grid box. The black line shows the historical values form 1979–2015 and the red line is the linear trend. The temperature at $T_{B}$ is the expected value in 2015 $\hat{E}y_{t_{0},s}$ from the linear regression model

The model error term $\varepsilon_{t,s}$ refers to the natural variability related to both time and space and it includes the prediction error naturally. Since models often have large biases in their natural variability (see,e.g., Hawkins and Sutton 2012; McKinnon et al. 2017; Chan et al. 2020; Zeppetello et al. 2020), we use the observations, i.e., the ERA5 reanalysis data to estimate it. The data include historical local temperature from 1979 to 2015 and are denoted by $\left(y_{1979,s},\ldots ,y_{2015,s}\right)$ for each grid box s. We subtract a linear trend from $\left(y_{1979,s},\ldots ,y_{2015,s}\right)$ to get the residuals and denote them by $\left({\hat{\varepsilon }}_{1979,s},\ldots ,{\hat{\varepsilon }}_{2015,s}\right)$. Figure 2 gives an illustration of the historical temperatures and linear trend for one grid box.

The linear trend is approximate, but not exact. To assess sensitivity to this methodological choice, we also carried out the analyses using a local polynomial (and thus nonlinear) trend rather than a linear trend for each grid bax. However, the results were almost indistinguishable, and so we chose to use the simpler linear trend approach.

For each grid box s, the histograms of the first- and second-order autocorrelations of the residuals, as well as the second-order partial autocorrelations, are close to zero. We assume that $\varepsilon_{t,s}\sim 𝒩\left(0,\sigma_{s}^{2}\right)$, and we estimate $\sigma_{s}$ by the sample standard deviation, namely

(11) $${\hat{\sigma }}_{s}=\sqrt{\frac{1}{36}\overset{2015}{\underset{t=1979}{\sum }}{\left({\hat{\varepsilon }}_{t,s}-\frac{1}{37}\overset{2015}{\underset{t=1979}{\sum }}{\hat{\varepsilon }}_{t,s}\right)}^{2}}.$$

For probabilistic projection of the future, we sample ${\hat{\varepsilon }}_{2016,s},\ldots ,{\hat{\varepsilon }}_{2100,s}$ independently from the normal distribution $𝒩\left(0,{\hat{\sigma }}_{s}^{2}\right)$.

#### Estimation of $Ey_{t_{0},s}$

We take $t_{0}=2015$ and estimate $Ey_{t_{0},s}$ by

(12) $$\hat{E}y_{t_{0},s}=y_{t_{0},s}-{\hat{\varepsilon }}_{t_{0},s},$$

where $y_{t_{0},s}$ is the historical local temperature from the ERA5 reanalysis data and ${\overset{ˆ}{\varepsilon }}_{t_{0},s}$ is the residual defined in the above paragraph. Specifically, we use the temperature $T_{B}$ (Fig. 2) as the estimate of $Ey_{t_{0},s}$.

#### Projections of local temperature

We make projections of local temperature using both the multi-pattern method and the single-pattern method. The multi-pattern method is based on Eq. (1) and the single-pattern method is based on Eq. (2). For the multi-pattern method, we draw 200 trajectories from the posterior distribution of $\left(a_{2016,i},\ldots ,a_{2100,i}\right)$ for each CMIP 5 model i (Liu and Raftery 2021). Given grid box s, we sample $\left({\overset{ˆ}{\varepsilon }}_{2016,s},\ldots ,{\overset{ˆ}{\varepsilon }}_{2100,s}\right)$ independently from the distribution $𝒩\left(0,{\overset{ˆ}{\sigma }}_{s}^{2}\right)$ and use (12) and (6) to estimate $\overset{ˆ}{E}y_{2015,s}$ and ${\overset{ˆ}{b}}_{s,i}$ respectively. We then get the forecast of local temperature $\left({\overset{ˆ}{y}}_{2016,s},\ldots ,{\overset{ˆ}{y}}_{2100,s}\right)$ by equation (1).

This procedure is implemented for each trajectory, and finally we obtain 200 trajectories of local temperature projections for each CMIP 5 model and each grid box s. We combine the 35 CMIP 5 models and get a total of 7000 trajectories of the local temperature projections $\left({\overset{ˆ}{y}}_{2016,s},\ldots ,{\overset{ˆ}{y}}_{2100,s}\right)$ in the last step. These 7000 trajectories are a sample from the predictive distribution of local average temperature over time in grid box s.

For the single-pattern method, we obtain 7000 trajectories of global averaged temperature projections $\left(a_{2016},\ldots ,a_{2100}\right)$ by first combining all the 35 CMIP 5 models together, and then using (2) to derive the 7000 trajectories of local temperature projections $\left({\overset{ˆ}{y}}_{2016,s},\ldots ,{\overset{ˆ}{y}}_{2100,s}\right)$.

### 2.3 Out-of-sample predictive validation

**Fig. 3:**
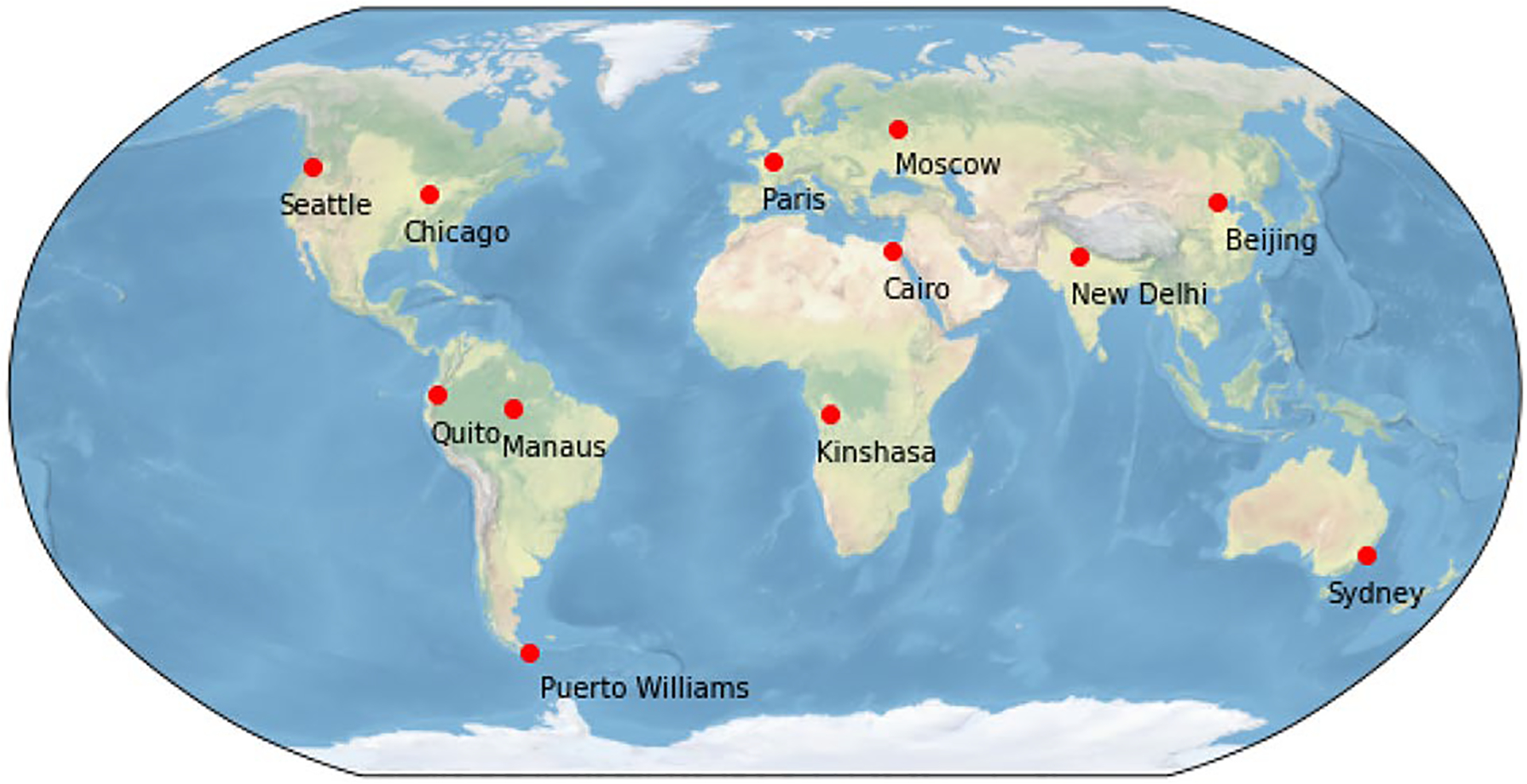
World map. Red dots are the cities we plot

**Fig. 4:**
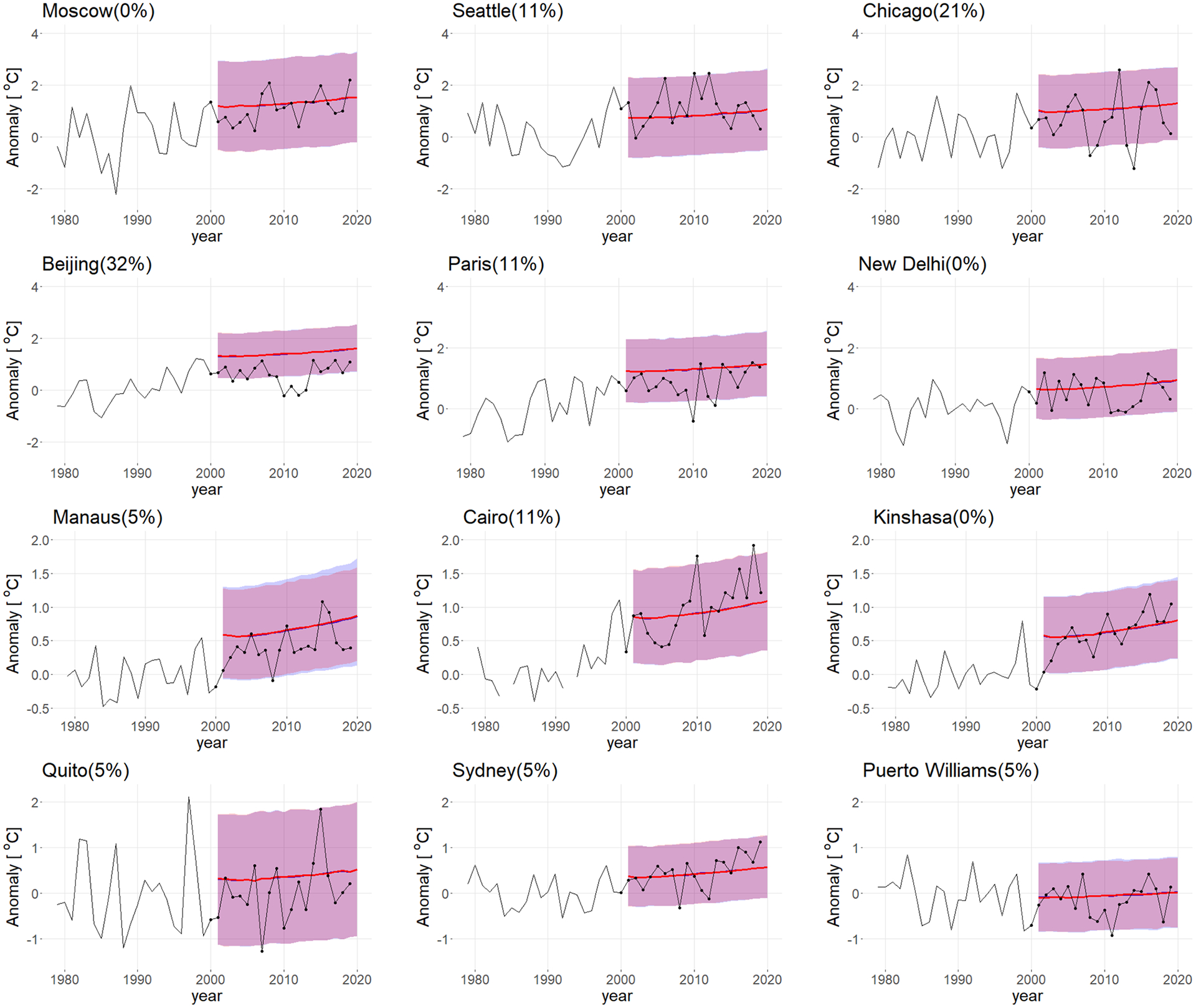
Out-of-sample predictive validation of model for temperature anomaly (in °C). Model estimated from data prior to 2000, predictions for 2001–2019. The red line is the median projection under the single-pattern method and the blue line is the median projection under the multi-pattern method. The two lines overlap since the median projections are very close. The red shaded region is the 90% prediction range for the single-pattern method and the blue shaded region is the 90% prediction range for the multi-pattern method. The black line is the historical temperature anomaly from 1979–2019 and the black dots are the historical values for the years 2000–2019. The percentage of dots outside the blue shaded region is marked after the city name

Out-of-sample predictive validation is a way to assess our statistical model. We fit the model using only data prior to 2000, make predictions for 2001–2019, and compare them with what actually happened. Table 1 gives the empirical coverage of the 90% and 95% prediction intervals for both multi-pattern and single-pattern methods for all gridboxes. We compute the empirical coverage by calculating the proportion of the actual values of 2001–2019 that lie in the prediction interval. The coverage for multi-pattern method is slightly larger than that for the single-pattern method since the former incorporates more variability, but they are both close to the nominal coverage. Figure 3 shows that almost all the black dots lie within the shaded regions for 12 cities (shown in Fig. 4), selected to illustrate the results for the regional end-of-century temperature projections over a wide range of environments (e.g. tropical, midlatitude, polar, maritime and continental locations). These results indicate that the model is reasonably well calibrated.

## 3 Results

### 3.1 Estimation results

**Fig. 5:**
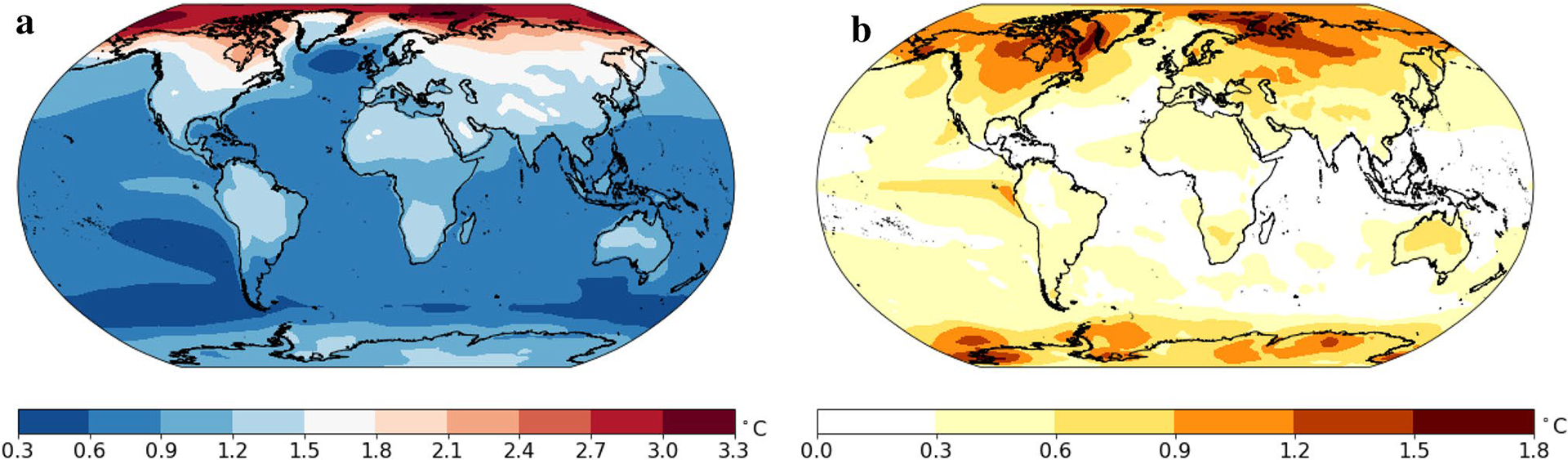
**a** Map of the spatial pattern $b_{s}$. **b** Map of the location-specific standard deviation, $\sigma_{s}$. Units are °C

Figure 5a shows the pattern of temperature change $b_{s}$ using the single-pattern scaling method on the output of the CMIP 5 simulations using historical data and forcing to 2100, and the RCP 8.5 emission scenario for the 21st century. Since the pattern is essentially scenario-independent (Tebaldi and Arblaster 2014), it is reasonable to just use the simulations of RCP 8.5 to estimate the common pattern, and the pattern of change for each model ${\overset{ˆ}{b}}_{s,i}$. Consistent with many studies dating back to Manabe et al. (1991), we find that high latitudes warm more than low latitudes, continents warm more than oceans and the Northern Hemisphere warms more than the Southern Hemisphere. These results are similar to those of Tebaldi and Arblaster (2014).

Figure 5b shows the amplitude of natural variability $\sigma_{s}$ that refers to the variations in annual averaged temperature caused by non-human forces. Natural variability is generally large in the polar regions and small in the tropical regions, except for the eastern tropical Pacific Ocean which experiences large variability due to the El Niño–Southern Oscillation (ENSO) phenomenon (Rasmusson and Carpenter 1982).

### 3.2 Projection results

#### Projections for the average of 2081–2100

**Fig. 6:**
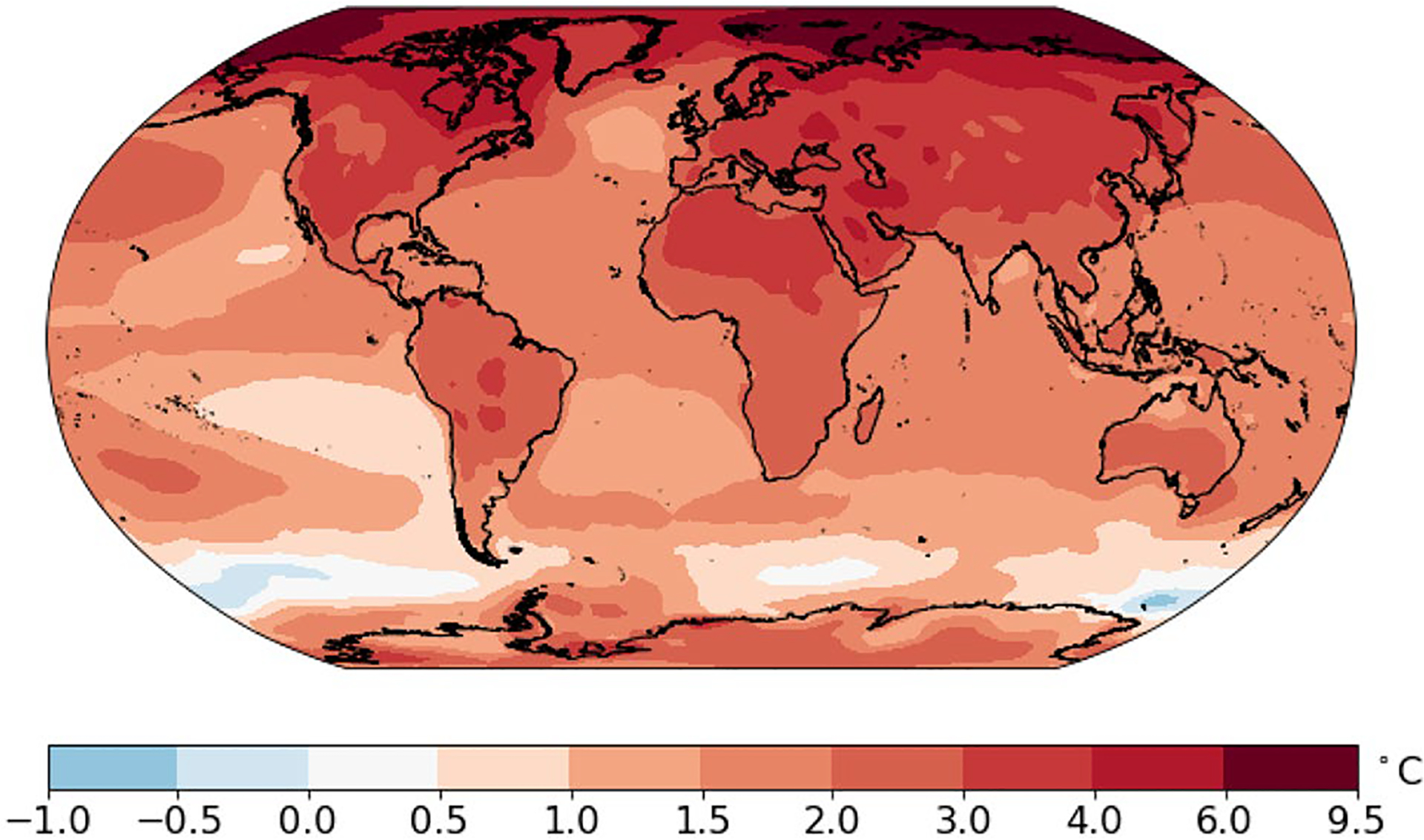
Median projections of the change in annual-averaged temperature for 2081–2100 compared to 1979–1998. Projections are based on the multi-pattern method. Units are °C

**Fig. 7:**
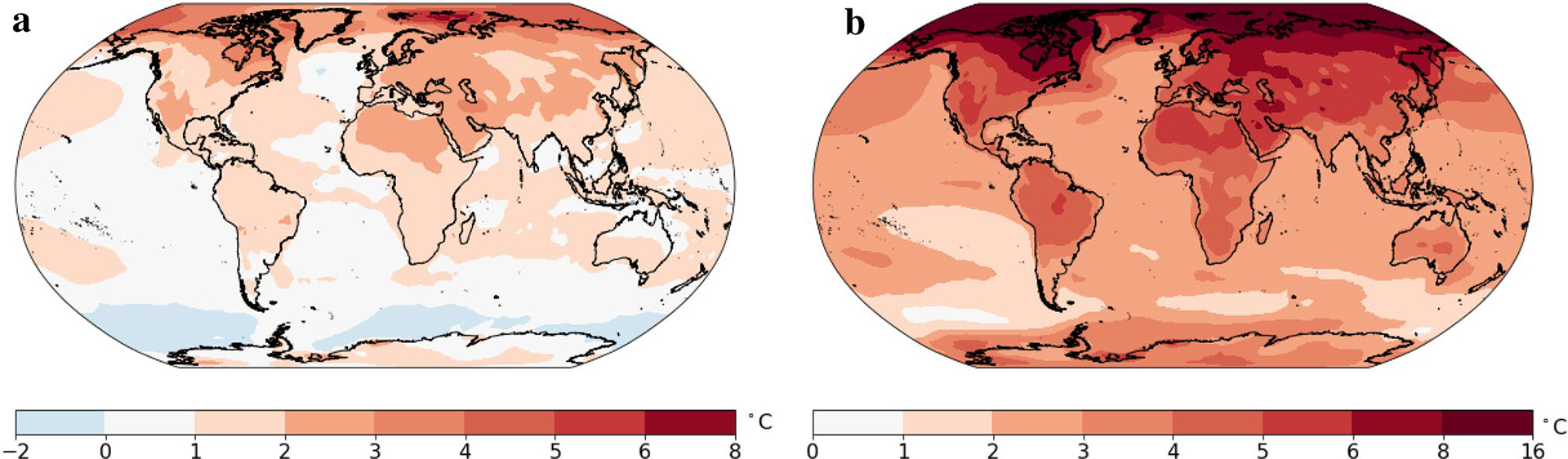
**a** 5th percentile for the projections of the change in annual-averaged temperature for 2081–2100 compared to 1979–1998. **b** 95th percentile for the projections of the change in annual-averaged temperature for 2081–2100 compared to 1979–1998. Projections are based on the multi-pattern method

**Fig. 8:**
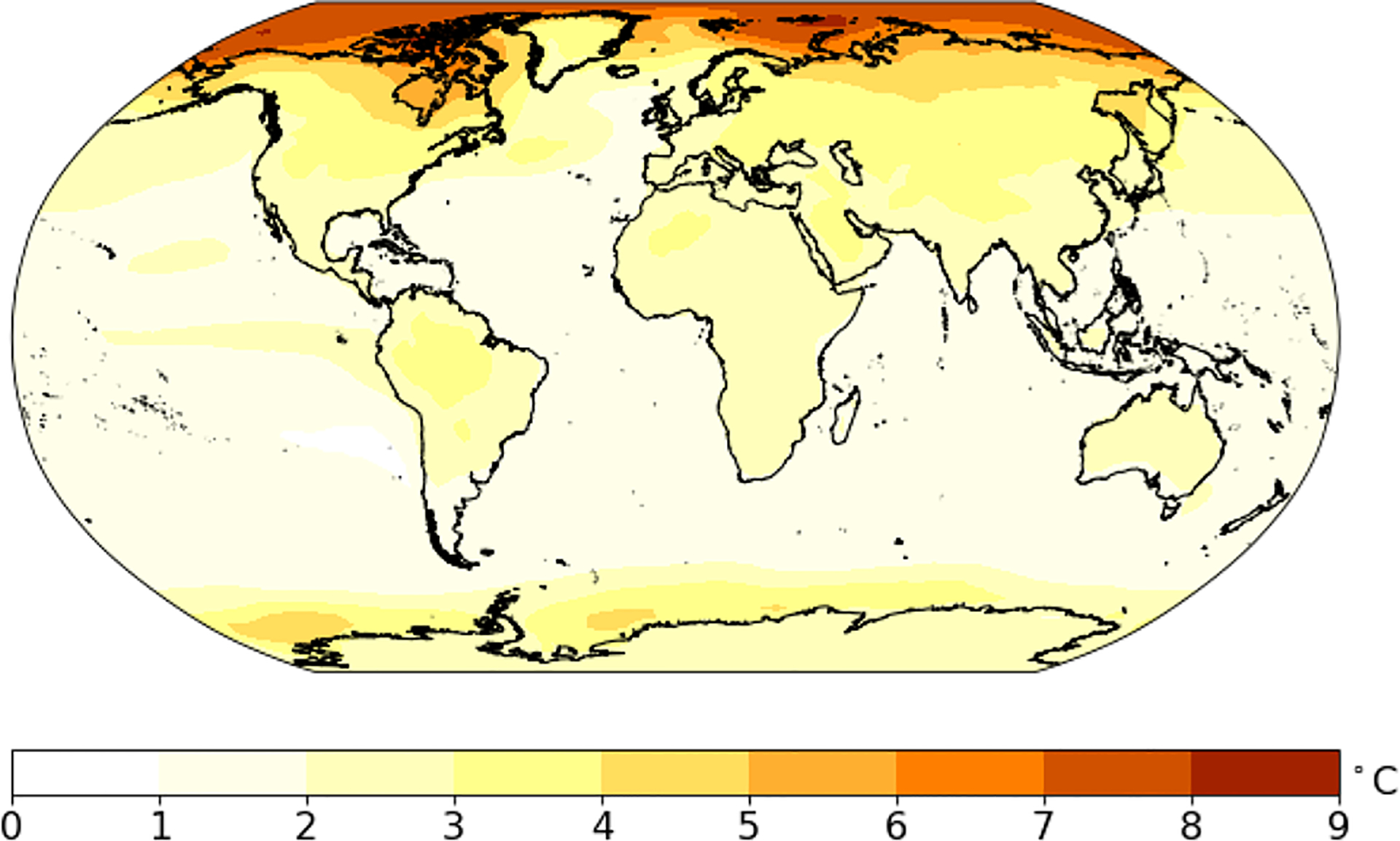
90% prediction range for average temperature of 2081–2100 (i.e., the difference between **a** and **b** in Fig. 7)

Figures 6, 7 and 8 show our projections of the average temperature anomaly for 2081–2100. The temperature anomaly is defined as the change in temperature compared to the 1979–1998 mean. Figures 10 and 11 show the results for a *single* (arbitrary) year at the end of this century (2100). We show only the projection results of the multi-pattern method because they incorporate the variability of patterns from different CIMP5 models. In fact, the results from the multi-pattern and single-pattern methods are very close (see, e.g., Fig. 13).

Figure 6 shows the median projections of the temperature anomaly for the average of 2081–2100 relative to the temperature at the end of the last century. The median temperature change exceeds 3 °C over almost all Northern Hemisphere land regions, and the median temperature increase is close to 9.5 °C in much of the Arctic.

Figures 7 and 8 show the lower and upper bounds of the likely range, or 90% prediction interval for the change in the 20-year average temperature. In the lower bound case shown in Fig. 7a, almost all regions will experience a temperature increase in the late twenty-first century compared to the late twentieth century. The only exceptions are the oceans near Antarctica and a small region in the North Atlantic. In this lower bound situation, the increase for the continents will be in the range 1–3 °C. In this optimistic but unlikely case, the temperature increases by more than 2 °C in the southwestern United States, Eastern and Northern Canada, West Asia, Northern Russia, Northern China, most of Europe, and North Africa. Figure 7b shows the upper bound (95th percentile) case. The increase over land is in the range 3–8 °C. The 95th percentile change exceeds 4 °C over most land areas in the Northern Hemisphere. The temperature increase for the Arctic could reach 16 °C in some regions.

Figure 8 shows the width of the 90% prediction interval, which characterizes the overall uncertainty including internal variation, model uncertainty, scenario uncertainty and also the prediction error variation (Hawkins and Sutton 2009). Generally, temperature projections in the higher latitudes are less certain than at low latitudes, and temperature projections over land are less certain than over oceans. For land regions, the width of the 90% prediction interval is 2–4 °C, while over oceans this difference is much smaller, at 1–2 °C.

**Fig. 9:**
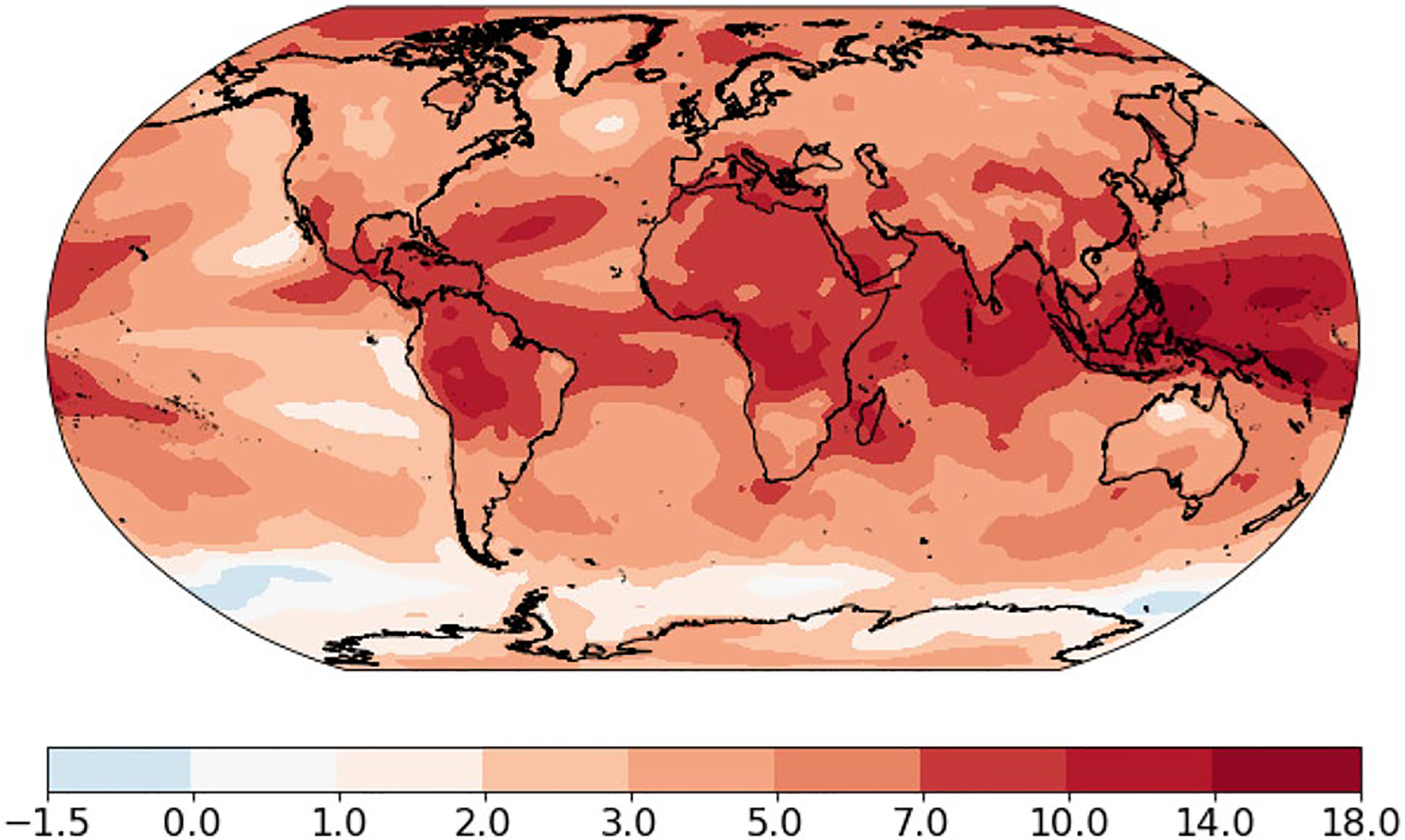
Median change in average temperature for 2081–2100 relative to the amplitude of natural variability

Figure 9 gives a measure of the signal-to-noise ratio for the change in the 20-year averaged temperature relative to the natural year-to-year variability. Generally, the tropical area has a large signal-to-noise ratio due to its small natural variability. The ratios for most of the land regions exceed 3 which means the temperature at the end of this century will exceed that of natural variability by at least a factor of 3.

#### Projections for an individual year at the end of the 21st century

**Fig. 10:**
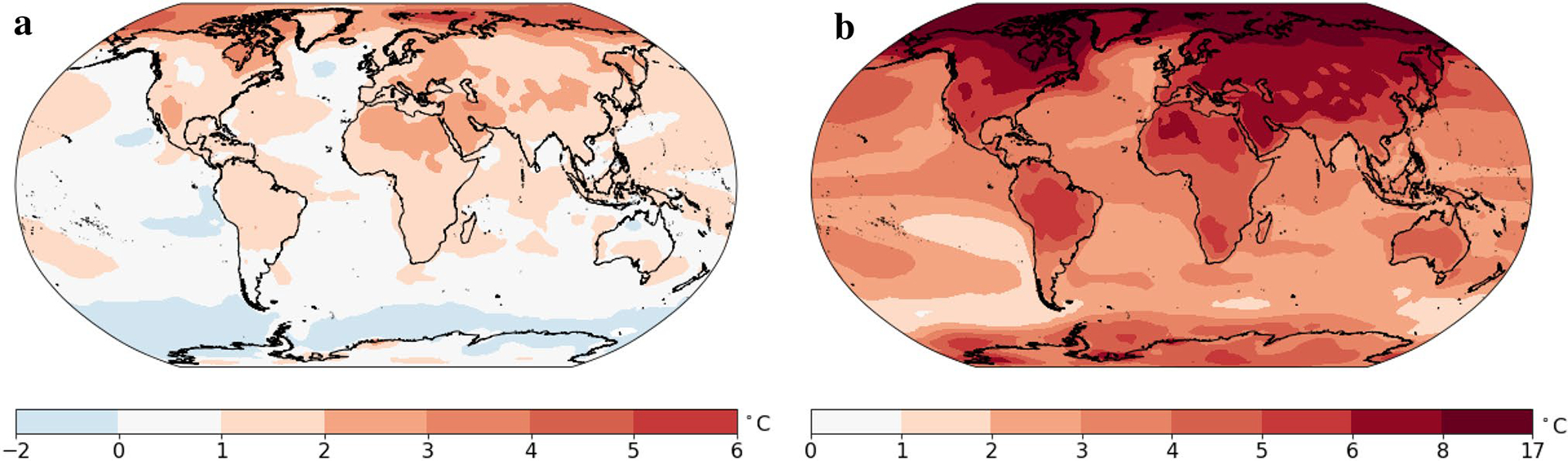
**a** 5th percentile of the predictive distribution of the change for 2100 compared to 1979–1998. **b** 95% percentile for the projections of the change for 2081–2100 compared to 1979–1998. Projections are based on the multi-pattern method

**Fig. 11:**
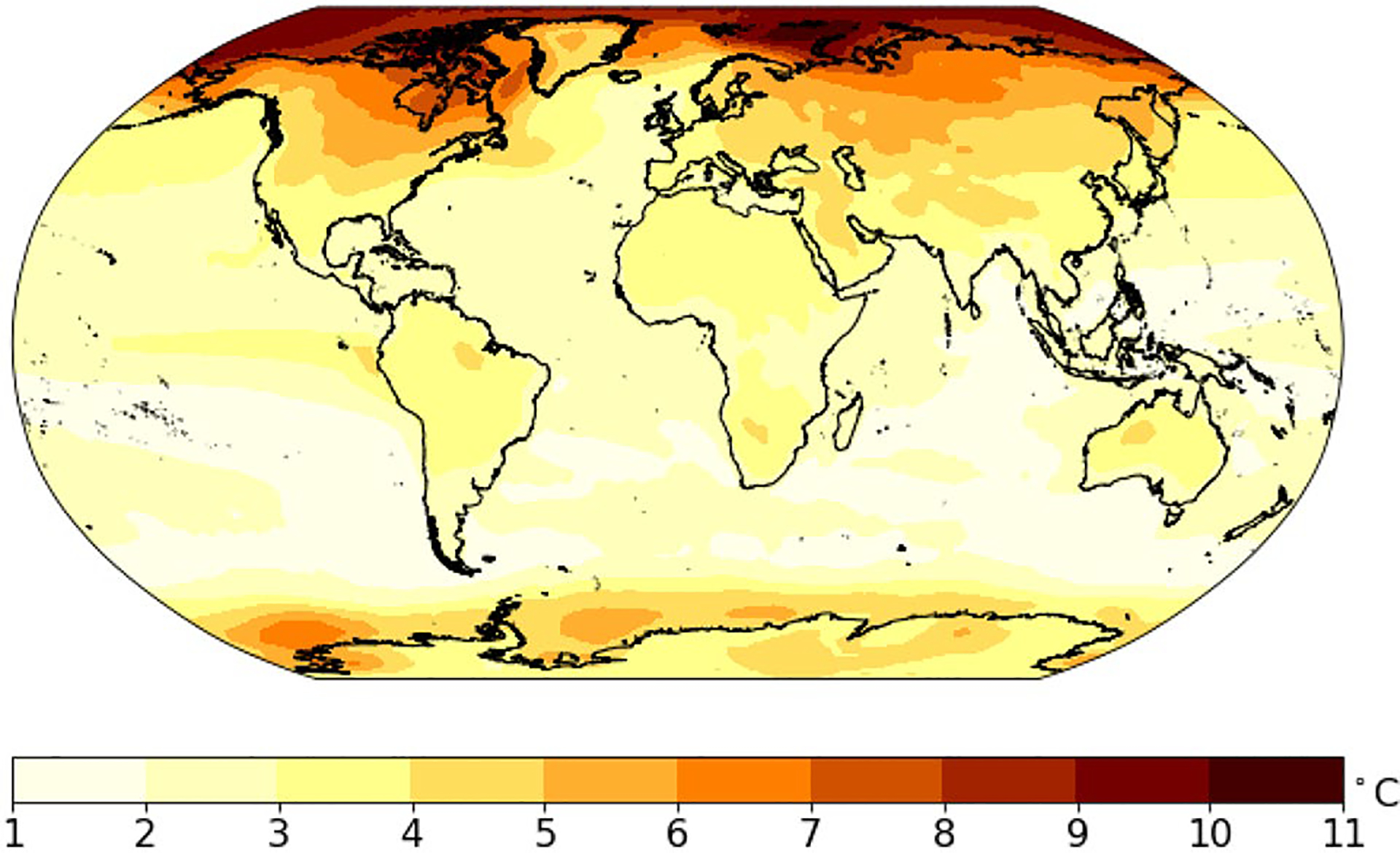
90% prediction range for temperature at 2100

**Fig. 12:**
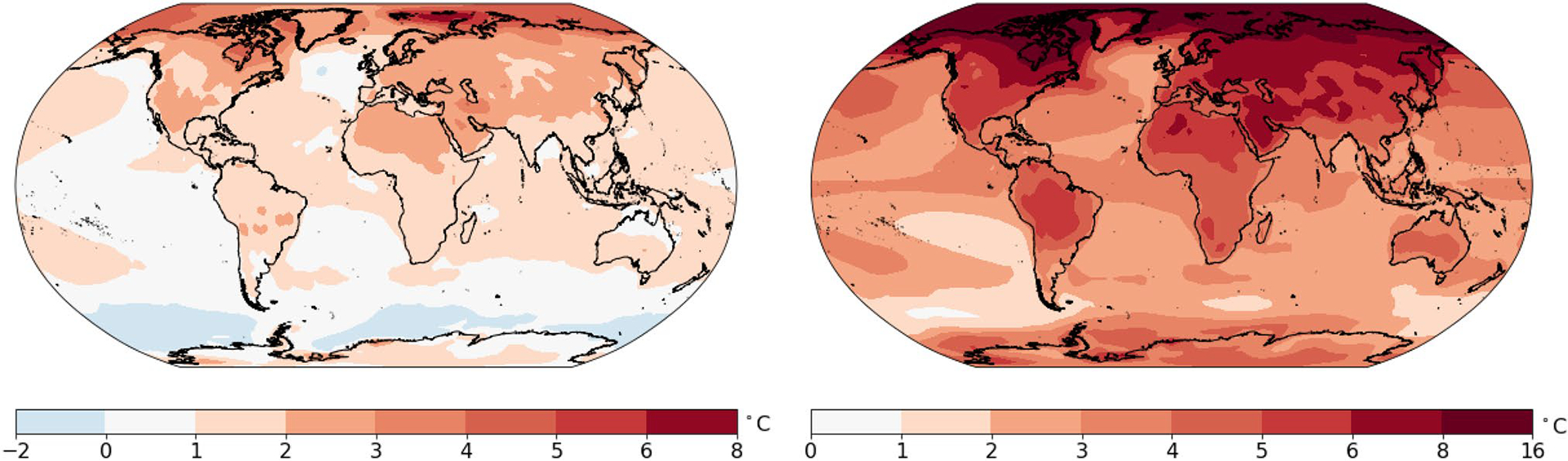
As in Fig. 10, but excluding the contribution of natural variability in the future projection

Figures 10, 11 and 12 show the resulting projections of temperature anomaly for a single (nominal) year at the end of the 21st century (i.e. the year 2100) under the multi-pattern method. Figure 10 shows the upper and lower bound. Liu and Raftery (2021) concluded that the probability of staying below 2 °C of global warming at 2100 is 5%. Figure 10a shows that the continents will have temperature increases in the range [1, 3] °C at a minimum (with probability 5% of being lower). Compared to Fig. 7a, the 5th percentile projected temperature increases for a single (nominal) year are slightly cooler—a consequence of much smaller amplitude natural variability on decadal time scales than on annual time scales. For the same reason, the 95th percentile projected temperature increases for a nominal year at the end of the 21st century are slightly greater than those for the 20-year average temperature projection. The range in the single year temperature projection at the end of the 21st century is shown in Fig. 11.

The impact of natural variability on the projections of a nominal year’s temperature at the end of the century is best seen by removing natural variability from the projections, which is done in Fig. 12. Removing natural variability reduces the spread in the projections of annual average temperature (cf. Figs 10, 12) and makes it very similar to the spread in 20-year averaged temperature (cf., Figs. 7, 12), which is a result of the preeminence of the uncertainty in climate sensitivity and emissions over natural variability in setting the spread in the probability of century-end temperature (consistent with results presented in Hawkins and Sutton 2009, 2012; Meehl et al. 2014).

#### Projections for different cities

**Fig. 13:**
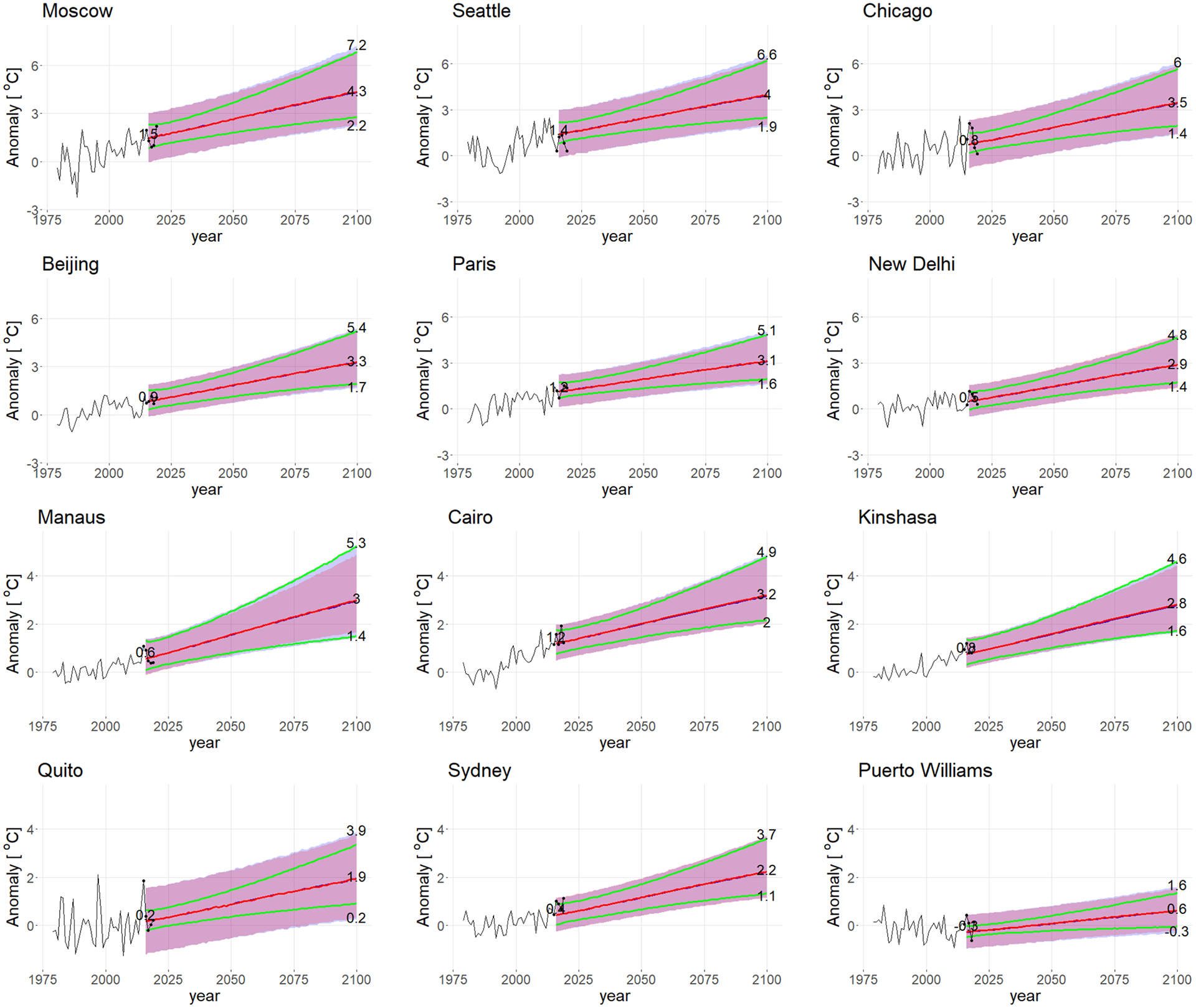
Projected annual-averaged temperature (in °C) for selected cities (see Fig. 3 for location). The blue and red lines (which are nearly on top of each other) denote the median projected temperature change using the multi-pattern method and the single pattern method, respectively; the blue and red shading denotes the 90 percentile range in the projected temperature change using the two methods. The green lines are the 5% and 95% quantile of the projections using the multi-pattern method, but after removing the natural variability $\varepsilon_{t,s}$. The black line is the historical temperature anomaly from 1979–2019 and the black dots are the historical values for 2015–2019. The numbers along the right side of each panel represent the median, 5% and 95% quantile of projected temperature using the multi-pattern method

Figure 13 shows the historical and projected anomalies for 12 selected cities. The greatest increases in median temperatures at the end of the century are for high latitude cities, where median temperature changes exceed 3 °C (see also Fig. 6). Temperature changes in the deep tropics are generally slightly less (see also Fig. 6).

The blue and red lines denote the median projected temperature change using the multi-pattern method and the single-pattern method, respectively; the blue and red shading denotes the 90 percentile range in the projected temperature change using the two methods. For all cities, the median and spread in the projections using the multi-pattern method are indistinguishable from the single-pattern method, as expected (see Tebaldi and Arblaster (2014)). At the beginning of the forecast period (2016), natural variability is a moderate contributor to the overall uncertainty in the extratropics and high-latitudes (e.g., Beijing, Moscow, Seattle, Chicago, Paris, and Puerto Williams) (Hawkins and Sutton 2009; Meehl et al. 2014, show similar results for selected emission scenarios). However, its contribution to the overall uncertainty falls rapidly and by the end of the century it accounts for very little of the uncertainty in the projections. Almost all of the prediction uncertainty at the end of the century stems from model uncertainty (i.e., climate sensitivity) and uncertainty about future emissions. Hawkins and Sutton (2009) showed a similar result for the 10-year average of the global mean temperature using the CMIP 3 projections.

Quito is somewhat of an exception. As a result of ENSO, Quito experiences relatively large natural variability for a tropical location—so much so that natural variability contributes moderately to the overall uncertainty in temperature throughout the century; natural variability still accounts for ~ 32% of the overall uncertainty in projected temperature in 2100.

Puerto Williams is a coastal city that lies in the Southern Ocean; it has the smallest temperature increase (0.6 °C) and prediction uncertainty of all the cities shown here. This is because of the very high heat capacity of the Southern Ocean (i.e., a very deep mixed layer), which mutes the surface temperature anomaly that results from natural variability in the surface energy budget and the temperature increase due to increasing CO_2_.

Finally, we note that the predictions for the period 2015–2019 largely lie within the 90% forecast interval projected temperatures only when natural variability is include in the projections. This underlines the importance of accounting for natural variability (the green lines in Fig. 13) when producing probabilistic forecasts for a given year.

## 4 Discussion

Previous work summarized in the Working Group 1 reports of the Intergovernmental Panel on Climate Change (IPCC) (Houghton et al. 2001; Solomon et al. 2007; Stocker 2014) gives a projection map of temperature change at 2100 but it is not fully probabilistic and statistically-based. We have proposed a probabilistic approach to give a spatial long-term forecast of average annual temperature. It aims to take account of all the major sources of uncertainty, including model uncertainty, scenario uncertainty and natural variability; it thus accounts for prediction error in a natural way. Our approach combines the pattern scaling techniques and the probabilistic forecast of global averaged temperature in Liu and Raftery (2021), and finally gives a probabilistic forecast of local temperature in a Bayesian framework using an MCMC sampling method. This yields a probability distribution for the future annual-average temperature in each year to 2100 and for each box in a regular latitude-longitude 2.5° grid.

We also analyze the sources of variability. Natural variability takes up a large portion of overall uncertainty in the short term for most of the area. Its contribution falls rapidly over time so that by 2100 the prediction uncertainty will come mostly from model uncertainty and uncertainty about future emissions. However, for typical tropical areas like Kinshasa and Manaus the natural variability is small so that it contributes throughout the projection. However, for Quito which is close to the South Pacific Ocean, it still contributes a great deal in 2100.

One limitation of our approach is we do not account for spatial correlation between the noise in different grid boxes. This does not affect the prediction results for each individual grid box, which remain of high quality and well calibrated. However, for aggregated forecasts for regions made up of multiple grid boxes, ignoring spatial correlation beyond what is accounted for in the climate models could lead to underestimation of uncertainty. This is a topic of future research.

In assessing the relative importance of the sources of uncertainty (i.e., natural variability, climate model sensitivity, emission scenario) to the total projection uncertainty, our approach follows that of Hawkins and Sutton (2009). Here, we update their results using the more recent CMIP 5 model projections, and extend them to include probability distributions for a single year. There are two notable differences in our approach. First, we use observations to estimate natural variability, avoiding the often significant biases in the natural variability simulated by the models (see also Hawkins and Sutton 2012). Second, in place of three equally weighted emission scenarios, we use fully probabilistic projections of atmospheric CO_2_ based on country-level projections of population, GDP per capita, and carbon intensity.

## Figures and Tables

**Table 1:** Out-of-sample predictive validation: empirical coverage (%) of prediction intervals from probabilistic local temperature forecasting method for all gridboxes. Model trained using data prior to 2000, and tested using data for 2001–2019

	Single-pattern method	Multi-pattern method
90%	86.8	87.5
95%	92.5	93.1

## Data Availability

The data on which the figures are based will be made publicly available at the time of publication.
